# Lifetime Cost-Effectiveness of Structured Education and Exercise Therapy for Knee Osteoarthritis in Australia

**DOI:** 10.1001/jamanetworkopen.2024.36715

**Published:** 2024-10-01

**Authors:** Sean Docking, Zanfina Ademi, Christian Barton, Jason A. Wallis, Ian A. Harris, Richard de Steiger, Rachelle Buchbinder, Natasha Brusco, Kirby Young, Marcella Ferraz Pazzinatto, Dylan Harries, Christopher J. Vertullo, Ilana N. Ackerman

**Affiliations:** 1 School of Public Health and Preventive Medicine, Monash University, Melbourne, Australia; 2 Centre for Medicine Use and Safety, Monash University, Melbourne, Australia; 3 La Trobe Sport and Exercise Medicine Research Centre, La Trobe University, Melbourne, Australia; 4 Cabrini Health, Malvern, Australia; 5 South West Sydney Campus, School of Clinical Medicine, UNSW Medicine and Health, UNSW Sydney, Liverpool, Australia; 6 Department of Surgery Epworth Healthcare, The University of Melbourne, Melbourne, Australia; 7 School of Primary and Allied Health Care, Monash University, Melbourne, Australia; 8 South Australian Health and Medical Research Institute, Adelaide, Australia; 9 Australian Orthopaedic Association National Joint Replacement Registry, Adelaide, Australia; 10 Menzies Health Institute, Griffith University, Southport, Australia

## Abstract

**Importance:**

Structured education and exercise therapy programs have been proposed to reduce reliance on total knee replacement (TKR) surgery and improve health care sustainability. The long-term cost-effectiveness of these programs is unclear.

**Objectives:**

To estimate the lifetime cost-effectiveness of implementing a national structured education and exercise therapy program for individuals with knee osteoarthritis with the option for future TKR compared with usual care (TKR for all).

**Design, Setting, and Participants:**

This economic evaluation used a life table model in combination with a Markov model to compare costs and health outcomes of a national education and exercise therapy program vs usual care in the Australian health care system. Subgroup, deterministic, and probabilistic sensitivity analyses were completed. A hypothetical cohort of adults aged 45 to 84 years who would undergo TKR was created.

**Exposure:**

Structured education and exercise therapy intervention provided by physiotherapists. The comparator was usual care where all people undergo TKR without accessing the program in the first year.

**Main Outcomes and Measures:**

Incremental net monetary benefit (INMB), with an incremental cost-effectiveness ratio threshold of 28 033 Australian dollars (A$) per quality-adjusted life-year (QALY) gained, was calculated from a health care perspective. Transition probabilities, costs, and utilities were estimated from national registries and a randomized clinical trial.

**Results:**

The hypothetical cohort included 61 394 individuals (53.9% female; 93.6% aged ≥55 years). Implementation of an education and exercise therapy program resulted in a lifetime cost savings of A$498 307 942 (US $339 922 227), or A$7970 (US $5537) per individual, and resulted in fewer QALYs (0.43 per individual) compared with usual care. At a population level, education and exercise therapy was not cost-effective at the lifetime horizon (INMB, −A$4090 [−US $2841]). Subgroup analysis revealed that the intervention was cost-effective only for the first 9 years and over a lifetime only in individuals with no or mild pain at baseline (INMB, A$11 [US $8]). Results were robust to uncertainty around model inputs.

**Conclusions and Relevance:**

In this economic evaluation of structured education and exercise therapy compared with usual care, the intervention was not cost-effective over the lifetime for all patients but was for the first 9 years and for those with minimal pain. These findings point to opportunities to invest early cost savings in additional care or prevention, including targeted implementation to specific subgroups.

## Key Points

**Question:**

What are the lifetime costs and benefits of a structured education and exercise therapy program for knee osteoarthritis if implemented nationally in Australia compared with total knee replacement?

**Findings:**

This economic evaluation in a hypothetical cohort of 61 394 adults aged 45 to 84 years found that a structured education and exercise therapy program was not cost-effective over a lifetime horizon but was cost-effective for the first 9 years. Targeted implementation of this program to individuals with lower baseline pain levels was cost-effective over the lifetime.

**Meaning:**

These findings suggest that a national structured education and exercise therapy program may deliver substantial cost savings, with greater efficiencies gained through targeting specific patient subgroups.

## Teaser

This economic evaluation estimates the lifetime cost-effectiveness of implementing a structured education and therapy program for knee osteoarthritis in Australian adults.

## Introduction

Knee osteoarthritis is an important public health issue, with the health and economic burden increasing through aging populations and growing reliance on surgical interventions.^1,2^ Contemporary clinical guidelines recommend that nonsurgical management options, including education and exercise therapy, be exhausted before total knee replacement (TKR) is considered.^3,4^ Yet, individuals with knee osteoarthritis who present to a general practitioner are 3 times more likely to be referred to an orthopedic surgeon than a physiotherapist.^5^ Future projections have estimated that 161 000 TKR procedures would be performed by 2030 at a cost of 3.4 billion Australian dollars (A$),^2^ with up to one-third of procedures regarded as inappropriate based on validated appropriateness tools (eg, for people with minimal symptoms or radiologic signs).^6,7^

International programs that incorporate guideline-based education and exercise therapy, such as the Good Life With Osteoarthritis: Denmark (GLA:D) program, have shown clinically promising results in improving health outcomes and short-term TKR avoidance when implemented in the Australian health system.^8,9,10^ However, it is unclear whether a structured education and exercise therapy program represents value for money compared with TKR. In a randomized clinical trial (RCT), Skou et al^10^ reported that a structured education and exercise therapy program with the option of later TKR was cost-effective compared with immediate TKR at 2 years. However, longer-term evaluation is crucial given that TKR has high upfront costs but long-lasting benefits.^11,12^ Furthermore, the results were sensitive to baseline characteristics (age, sex, baseline costs, and quality-adjusted life-years [QALYs]), suggesting that the cost-effectiveness of these interventions may differ in certain subgroups.^10^

Structured education and exercise therapy programs are poorly funded by the Australian public health system and private health insurers. With financial costs a recognized barrier to access,^13,14^ there is a need to leverage national registry data to explore the cost-effectiveness of funding a structured education and exercise therapy program at a national level prior to TKR over a longer time horizon.

The aim of this study was to evaluate whether structured education and an exercise therapy program delivered to individuals with knee osteoarthritis with the option for future TKR is cost-effective across the lifespan compared with TKR in the first year from a health system perspective.

## Methods

### Study Design

This economic evaluation is reported according to the 2022 Consolidated Health Economic Evaluation Reporting Standards (CHEERS) checklist.^15^ Patient consent was obtained for the Australian Orthopaedic Association National Joint Replacement Registry (AOANJRR) Patient-Reported Outcomes Program and GLA:D Australia Registry. Ethical approval was obtained through the Monash University Human Research Ethics Committee to access these deidentified data.

We developed a life table model in combination with a Markov model to compare a structured education and exercise therapy program with the option for future TKR vs TKR in the first year (usual care). A Markov model uses mean participant data to replicate the clinical context where individuals can transition between different health states. The lifetime model, designed using annual cycles, comprised 4 health states: no or mild pain, moderate pain, severe or extreme pain, and dead. The 3 alive health states were solely defined by participant responses to the pain domain within the 5-level EuroQuol-5 Dimension (EQ-5D-5L) instrument. Individuals start in 1 of 3 alive health states, with dead as the absorbing health state. Half-cycle corrections were made, with an annual discount rate of 5% applied to costs and outcomes.^16^ Model inputs, their sources, and parameter distribution are summarized in Table 1 and eTables 1 to 11 in Supplement 1.^10,17,18,19,20,21,22,23^ The model was built using Microsoft Excel, version 16.87 (Microsoft Corporation).

**Table 1.** Model Input Probabilities, Utilities, and Costs

Variable	Base case	Source	Parameter distribution
Population	Australian population 2022 (eTable 1 in Supplement 1)	Australian Bureau of Statistics,^17^ 2023	NA
Proportion eligible for TKR	Age- and sex-specific (eTable 1 in Supplement 1)	Australian Institute of Health and Welfare,^18^ 2023	NA
Baseline proportion of health states	Age- and sex-specific (eTable 2 in Supplement 1)	AOANJRR^a^	Dirichlet
All-cause mortality	Age- and sex-specific (eTable 3 in Supplement 1)	Australian Bureau of Statistics,^19^ 2023	Log-normal
Excess mortality following TKR	Based on intervals post TKR (eTable 4 in Supplement 1)	Harris et al,^20^ 2019	Log-normal
Undergoing TKR following nonsurgical management, proportion (95% CI)		Skou et al,^10^ 2020	Beta
First y	0.26 (0.14-0.38)		
Subsequent y	0.08 (0.03-0.21)		
Probability of TKR revision	Age- and sex-specific and year after primary TKR (eTable 5 in Supplement 1)	AOANJRR,^21^ 2022	Beta
Transition probabilities between health states following primary TKR	Age- and sex-specific (eTable 6 in Supplement 1)	AOANJRR^a^	Dirichlet
Transition probabilities between health states following nonsurgical management	Age- and sex-specific (eTable 6 in Supplement 1)	GLA:D Australia Registry^a^	Dirichlet
**Costs, 2022 A$ ($)**			
Primary TKR^b^		Independent Hospital Pricing Authority,^22^ 2021	Gamma
Public	20 955 (14 557)		
Private	25 435 (17 670)		
Weighted mean	24 607 (17 095)		
Revision TKR^b^		Independent Hospital Pricing Authority,^22^ 2021	Gamma
Public	43 233 (30 034)		
Private	43 078 (29 926)		
Weighted mean	43 125 (29 959)		
Nonsurgical management^c^	1500 (1042)	Ackerman et al,^23^ 2020	Gamma

Abbreviations: A$, Australian dollars; AOANJRR, Australian Orthopaedic Association National Joint Replacement Registry; GLA:D, Good Life With Osteoarthritis: Denmark; NA, not applicable; TKR, total knee replacement.

^a^ Individual patient data requested.

^b^ Sensitivity analysis–assessed SE of 15% on surgical costs.

^c^ Sensitivity analysis–assessed range of A$750 ($521) to A$3000 ($2084) on nonsurgical costs.

### Model Population

The model population comprised adults aged 45 to 84 years who would otherwise undergo TKR in Australia (eligible patients), excluding those undergoing partial knee replacements, as they are infrequently performed.^21^ To calculate the size of our hypothetical cohort, we obtained age- and sex-specific rates of TKR for osteoarthritis in 2020-2021^18^ and 2022^17^ Australian population data (Table 1; eTable 1 in Supplement 1). We estimated that 61 394 Australians underwent primary TKR in 2022.

Individuals were categorized into health states based on the EQ-5D-5L pain domain. The baseline proportion of individuals in each health state was calculated from individual-level patient-reported outcome measures from the AOANJRR. Preoperative patient-reported outcome measures have been collected since mid-2018, with data available for 73.5% of procedures.^24^ For this study, data were obtained for 9889 individuals who underwent primary TKR for osteoarthritis from August 7, 2018, to December 31, 2021 (eTable 2 in Supplement 1). The baseline proportions of individuals in each health state based on age and sex strata are provided in eTable 3 in Supplement 1.

All-cause mortality rates were derived from Australian population life tables (eTable 4 in Supplement 1). Mortality risk following TKR was adjusted given evidence of fewer deaths in the first 8 to 9 years following TKR and more deaths after 12 years^20^ (eTable 5 in Supplement 1).

As osteoarthritis is a degenerative disease that may influence multiple joints (contralateral knee, hip, or shoulder), we took a conservative approach and assumed that the rate of contralateral TKR surgery or replacement surgery at other joints would not be influenced by the intervention.

### Transition Probabilities

Transition probabilities following TKR were derived from AOANJRR data, stratified by age and sex (eTable 6 in Supplement 1). The AOANJRR collects postoperative outcomes at 6 months as most clinical improvement occurs during this period.^25^ Transition probabilities following education and exercise therapy were obtained from the GLA:D Australia Registry at 12 months (eTable 7 in Supplement 1). As this database includes all individuals with osteoarthritis who participated in the GLA:D program, we restricted our analysis to those who were (1) on a waiting list for a joint replacement or surgical opinion, (2) reported that they were in so much trouble and pain that they wanted surgery, or (3) had a Knee Injury and Osteoarthritis Outcome Score-12 summary score 54.6 or less. The Knee Injury and Osteoarthritis Outcome Score-12 summary score threshold of 54.6 aligns with the preoperative score plus 1 SD from 4010 individuals who underwent primary TKR for osteoarthritis in Australia.^26^

The likelihood of revision surgery, by age and sex, was obtained from the 2022 AOANJRR annual report (eTable 8 in Supplement 1).^21^ Likelihood of revision surgery may be slightly overestimated as death was censored as a noninformative factor rather than as a competing risk. The likelihood of TKR 2 years after a structured education and exercise therapy program was obtained from an RCT of 100 participants (Table 1).^10^ We assumed a consistent rate of TKR in the second and subsequent years following the structured education and exercise therapy program (Table 1). We also assumed that program effectiveness in delaying or avoiding TKR was similar across health states and age and sex strata. Data on race and ethnicity were not available. Uncertainty in this model input was assessed in a 1-way sensitivity analysis.

### Utilities

Health-related quality-of-life utilities were calculated using published Australian EQ-5D-5L value sets.^27^ Baseline utilities for each health state were calculated from preoperative EQ-5D-5L scores for individuals undergoing TKR (eTable 9 in Supplement 1). Postintervention utilities for TKR were calculated using AOANJRR 6-month postoperative EQ-5D-5L scores (eTable 10 in Supplement 1). To account for higher baseline utilities in GLA:D Australia Registry patients, we calculated the incremental utility and disutility based on the transition between health states for education and exercise therapy (eTable 11 in Supplement 1). Based on data for 268 individuals undergoing revision TKR in the AOANJRR, a disutility of 0.28 was applied in the cycle prior to TKR revision and a disutility of 0.10 for all subsequent cycles (an SE of 25% was assumed).

### Costs

The model estimated only direct costs from a health system perspective; all costs are presented in 2022 Australian dollars and converted into US dollars ($0.6947 for A$1^28^) (Table 1). Primary TKR cost in public hospitals was calculated using the weighted mean of costs for minor and major complexity procedures.^22^ Primary TKR cost in private hospitals was obtained from the websites of the 3 largest private health insurers and averaged.^29,30,31^ The mean cost of primary TKR, adjusting for the proportion of procedures performed in public and private hospitals, was A$24 607 ($17 095). Given no published data on revision TKR costs in private hospitals, we added 20% to the revision TKR cost in public hospitals^23^ based on expert clinician advice (I.A.H. and R.d.S.), with the mean cost of revision TKR estimated at A$43 125 ($29 959).

We estimated the cost of a 12-week structured education and exercise therapy program based on earlier methods (Table 1).^23^ While a 6-week program has been implemented in Australia (estimated cost of A$1000 [$695]), we chose to incorporate the costs of a 12-week program in line with the original trial.^10^

We did not incorporate any other health care costs outside the base case interventions as no difference in year 2 costs were observed in the RCT by Skou et al.^10^ This assumption was tested in the 1-way sensitivity analysis by varying these costs by A$500 ($347) annually for 5 years after primary TKR or education and exercise therapy.^32^

### Statistical Analysis

A cost-utility analysis was performed to calculate lifetime incremental costs and QALYs (number of life-years multiplied by utility value). As we anticipated that costs and QALYs would be lower after a structured education and exercise therapy program, we calculated the incremental net monetary benefit (INMB) with an incremental cost-effectiveness ratio (ICER) threshold of A$28 033 ($19 475) per QALY gained. This threshold is based on empirical estimates of opportunity costs for decisions to fund new health technologies.^33^ A positive INMB value indicates that a structured education and exercise therapy program is cost-effective (eg, cost savings are greater than the QALY losses), whereas a negative INMB value indicates that usual care is cost-effective (eg, cost savings do not outweigh the QALY losses). Subgroup analyses were conducted for each baseline health state, age and sex stratum, and hospital type (public vs private).

A 1-way deterministic sensitivity analysis was performed on all model inputs to ascertain the outcomes of input uncertainty and the robustness of model estimates. Probabilistic sensitivity analysis was performed by running 1000 Monte Carlo simulations to obtain 95% uncertainty intervals for INMB. The results are presented on a cost-effectiveness plane and cost-effectiveness acceptability curve across a range of ICER thresholds. All analyses were performed using Microsoft Excel, version 16.87.

## Results

### Base Case

**Figure 1.**
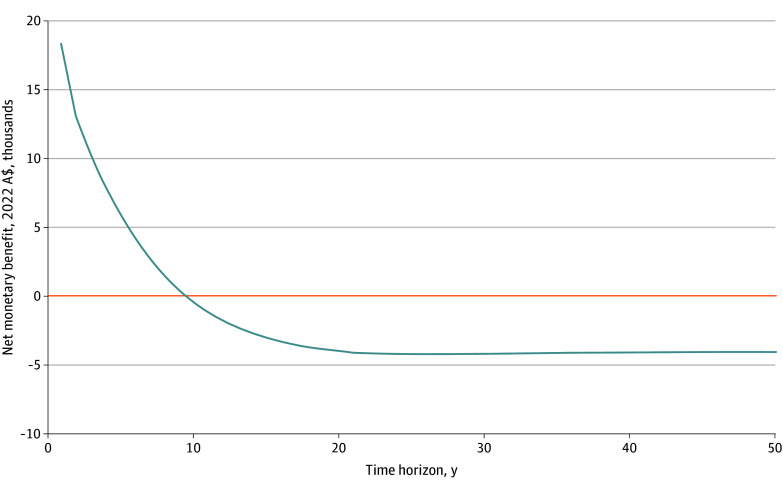
Cost-Effectiveness of Structured Education and Exercise Therapy vs Usual Care Over Time Horizontal orange line indicates the incremental cost-effectiveness threshold (incremental net monetary benefit, 0 Australian dollars [A$]).

The hypothetical cohort included 61 394 adults aged 45 to 84 years (53.9% females and 46.1% males; 93.6% aged ≥55 years). Implementation of a national education and exercise therapy program for knee osteoarthritis prior to TKR was estimated to produce a cost savings of A$489 307 942 ($339 922 227), or A$7970 ($5537) per person, over the lifetime horizon compared with usual care (Table 2). This estimate was derived from 19.5% individuals avoiding primary TKR and 14 418 fewer TKRs (11 995 fewer primary TKRs and 2423 fewer revision TKRs). There was no difference in total life-years between intervention and comparator. The education and exercise therapy program resulted in 0.43 fewer QALYs per person compared with usual care over the lifetime horizon. The INMB of −A$4090 (−$2841) indicated that a structured education and exercise therapy program would not be cost-effective over the lifetime horizon (Table 2). Structured education and exercise therapy was only cost-effective for the first 9 years of the time horizon (Figure 1; eTable 12 in Supplement 1).

**Table 2.** Nonsurgical Management Program vs Usual Care for Knee Osteoarthritis Over a Lifetime Horizon and Subgroup Analysis Based on Baseline Health State, Age, and Sex

Variable	Cost, 2022 A$ ($)		Incremental cost difference, 2022 A$ ($)	Total QALYs		Incremental QALY difference	INMB, 2022 A$ ($)
	Nonsurgical	Usual care		Nonsurgical	Usual care		
Base case (n = 61 394)							
Total	1 092 713 293 (759 107 924)	1 582 021 235 (1 099 030 152)	−489 307 942 (−339 922 227)	624 489	650 902	−26 412	−4090 (−2841)
Per person	17 798 (12 364)	25 768 (17 901)	−7970 (−5537)	10.17	10.60	−0.43	
**Baseline health state**							
No or mild pain (n = 12 473)							
Total	215 267 310 (149 546 200)	317 132 491 (220 311 942)	−101 865 181 (−70 765 741)	128 513	132 142	−3629	11 (8)
Per person	17 259 (11 990)	25 426 (17 663)	−8167 (−5674)	10.30	10.59	−0.29	
Moderate pain (n = 30 706)							
Total	546 735 808 (379 817 366)	790 850 027 (549 403 514)	−244 114 220 (−169 586 149)	316 909	330 169	−13 260	−4156 (−2887)
Per person	17 805 (12 369)	25 755 (17 892)	−7950 (−5523)	10.32	10.75	−0.43	
Severe or extreme pain (n = 18 215)							
Total	330 710 175 (229 744 359)	474 038 717 (329 314 697)	−143 328 542 (−99 570 338)	179 067	188 591	−9523	−6788 (−4716)
Per person	18 156 (12 613)	26 025 (18 080)	−7869 (−5467)	9.83	10.35	−0.52	
**Age group, y**							
45-54 (n = 3943)							
Total	91 483 696 (63 553 724)	121 227 208 (84 216 541)	−29 743 512 (−20 662 818)	51 226	54 724	−3498	−17 326 (−12 036)
Per person	23 202 (16 118)	30 745 (21 359)	−7543 (−5240)	12.99	13.88	−0.89	
55-64 (n = 16 567)							
Total	326 072 933 (226 522 867)	447 603 176 (310 949 926)	−121 530 243 (−84 427 060)	200 279	207 567	−7288	−4996 (−3471)
Per person	19 682 (13 673)	27 018 (18 769)	−7336 (−5096)	12.09	12.53	−0.44	
65-74 (n = 26 240)							
Total	457 322 473 (317 701 922)	660 314 256 (458 720 314)	−202 991 783 (−141 018 392)	268 203	278 403	−10 200	−3161 (−2196)
Per person	17 428 (12 107)	25 164 (17 481)	−7736 (−5374)	10.22	10.61	−0.39	
75-84 (n = 14 644)							
Total	217 834 191 (151 329 413)	352 876 596 (245 143 371)	−135 042 405 (−93 813 959)	104 781	110 208	−5427	−1166 (−810)
Per person	14 875 (10 334)	24 097 (16 740)	−9222 (−6407)	7.16	7.53	−0.37	
**Sex**							
Female (n = 33 069)							
Total	606 179 084 (421 112 610)	850 139 140 (590 591 661)	−243 960 056 (−169 479 051)	342 503	358 205	−15 702	−5934 (−4122)
Per person	18 331 (12 735)	25 708 (17 859)	−7377 (−5125)	10.36	10.83	−0.47	
Male (n = 28 325)							
Total	486 534 209 (337 995 315)	731 882 095 (508 438 492)	−245 347 886 (−170 443 176)	281 987	292 696	−10 710	−1938 (−1346)
Per person	17 177 (11 933)	25 839 (17 950)	−8662 (−6018)	9.96	10.33	−0.38	
**Hospital type**							
Public (n = 16 514)							
Total	271 856 461 (188 858 684)	390 514 531 (271 290 445)	−118 658 069 (−82 431 761)	166 920	174 342	−7422	−5414 (−3761)
Per person	16 462 (11 436)	23 647 (16 428)	−7185 (−4991)	10.11	10.56	−0.45	
Private (n = 44 878)							
Total	820 594 644 (570 067 099)	1 191 065 311 (827 433 072)	−370 470 667 (−257 365 972)	459 045	477 586	−18 541	−3327 (−2311)
Per person	18 285 (12 703)	26 540 (18 437)	−8255 (−5735)	10.23	10.64	−0.41	

Abbreviations: A$, Australian dollars; INMB, incremental net monetary benefit; QALY, quality-adjusted life-year.

### Subgroup Analyses

Results from subgroup analyses are presented in Table 2. Cost savings were similar when evaluating each baseline health state separately. When restricted to individuals with no or mild pain at baseline, the structured education and exercise therapy program was cost-effective (INMB, A$11 [$8]) as between-group incremental QALY losses were lower compared with the base case (−0.29 vs −0.43). The intervention was not cost-effective over the lifetime horizon when restricted to individuals with moderate pain or severe or extreme pain at baseline (INMB, −A$4156 [−$2887] and −A$6788 [−$4716], respectively).

The INMB for the intervention increased with older age categories (45-54 years, −A$17 326 [−$12 036]; 55-64 years −A$4996 [−$3471]; 65-74 years, −A$3161 [−$2196]; 75-84 years, −A$1166 [−$810]), yet usual care remained the cost-effective option for all age groups (Table 2). Younger age groups accumulated greater QALY gains from usual care (TKR in first year) over more years, resulting in greater incremental QALY losses compared with the structured education and exercise therapy. The greatest cost savings were observed in the 75- to 84-year age group as individuals died before requiring TKR. The INMB was higher for males than females (−A$1938 [−$1346] vs −A$5934 [−$4122], respectively), likely driven by more males having no or mild pain at baseline (eTable 2 in Supplement 1).

Usual care remained the cost-effective option in both public and private hospitals. However, a larger negative INMB was evident for public hospitals vs private hospitals (−A$5414 [−$3761] vs −A$3327 [−$2311], respectively) (Table 2) due to smaller between-group QALY losses in private hospitals through a greater proportion of individuals who had no or slight pain at baseline.

### Deterministic Sensitivity Analysis

One-way sensitivity analysis results are presented in the eFigure in Supplement 1. While uncertainty in health-related quality of life following structured education and exercise therapy greatly influenced cost-effectiveness, the model was robust to all other model inputs.

### Probabilistic Sensitivity Analysis

**Figure 2.**
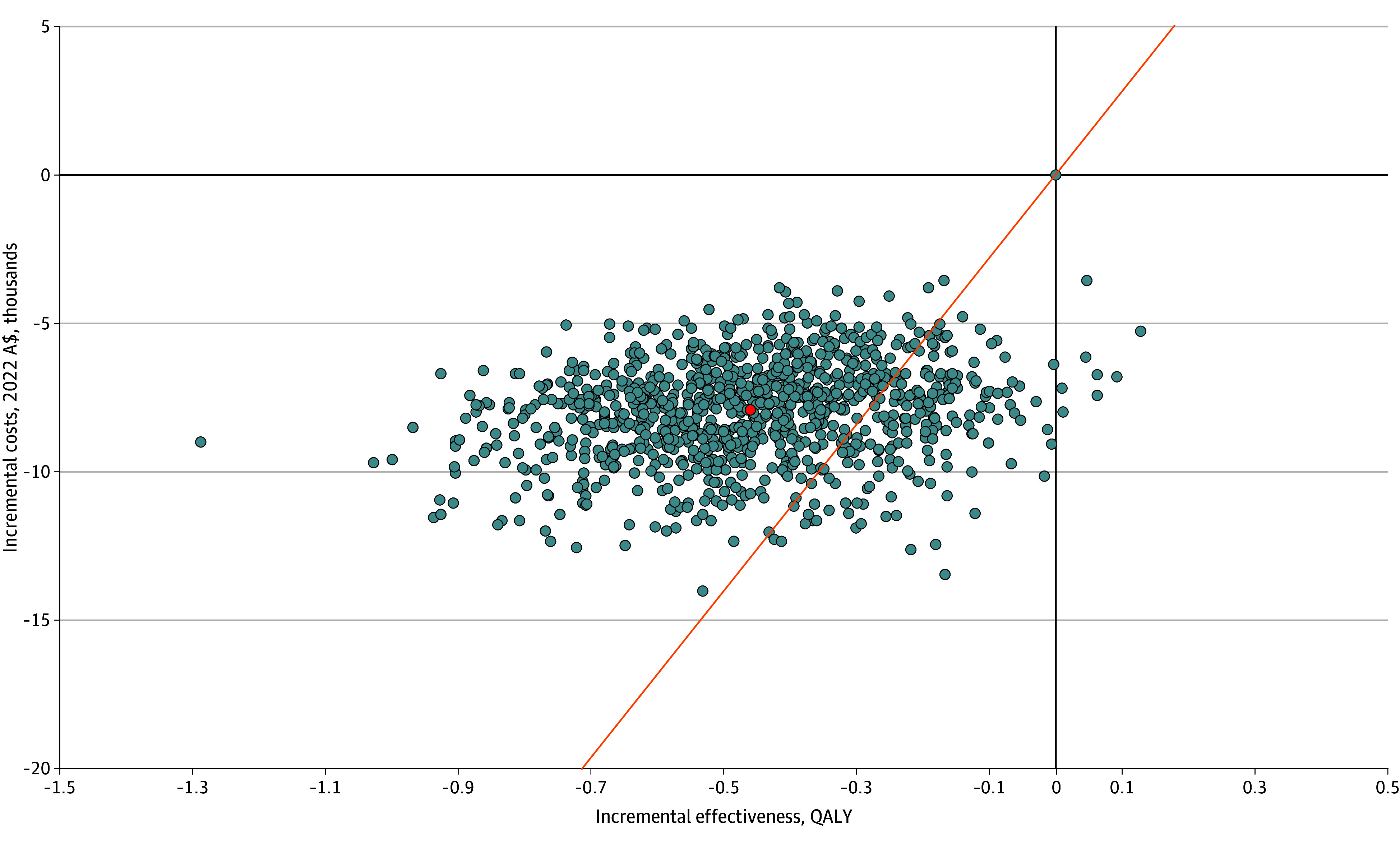
Cost-Effectiveness Plane for the Base Case Analysis Each blue dot represents a model iteration, and the red dot represents the mean cost-effectiveness of the 1000 iterations. The orange line represents the incremental cost-effectiveness threshold (28 033 Australian dollars [A$] per quality-adjusted life-year [QALY] gained). Blue dots below the orange line indicate that the model iteration was cost-effective.

**Figure 3.**
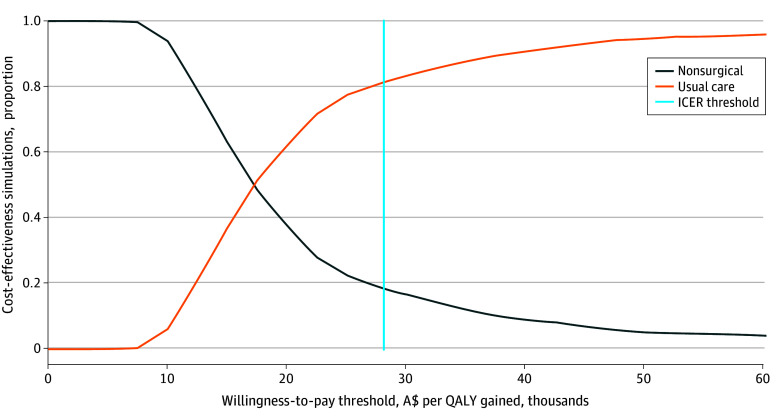
Cost-Effectiveness Acceptability Curve The blue vertical line indicates the base case incremental cost-effectiveness threshold (28 033 Australian dollars [A$] per quality-adjusted life-year [QALY] gained).

The cost-effectiveness plane (Figure 2) shows that education and exercise therapy were associated with lower costs and smaller QALY gains in 99.2% of simulations, with a mean INMB of −A$4897 (95% CI, −A$5229 to −A$4564) (−$3402; 95% CI, −$3633 to −$3171). The cost-effectiveness acceptability curve (Figure 3) shows that the nonsurgical management program was cost-effective in 18.6% of simulations at an ICER threshold of A$28 033 per QALY gained.

## Discussion

In this economic analysis, we found that a national structured education and exercise therapy program with the option for future TKR would produce substantial cost savings but would not be cost-effective over the lifetime horizon. Our results suggest that structured education and exercise therapy is cost-effective for the first 9 years and over the lifetime in individuals with low pain levels at baseline. These findings highlight the need for considered approaches that improve the sustainability of care and ensure that the right patient gets the right care at the right time.

Our results are consistent with previous studies despite important methodological differences.^11,34,35,36,37^ Previous models comparing TKR and nonsurgical management did not incorporate the likelihood of individuals undergoing TKR following nonsurgical treatment.^11,35,36^ This limitation is important given that the 2 interventions are not directly substitutable in clinical practice. Three previously published models^34,37,38^ assessed the cost-effectiveness of delaying TKR with nonsurgical management but did not consider that TKR may not be needed in a subset of the population.^6,7^ Our analysis, based on 2-year RCT outcomes,^10^ explicitly addresses these methodological issues.

Our results contrast somewhat with those of the RCT, as the authors reported that more intensive nonsurgical care (still centered on education and exercise therapy) was cost-effective.^10^ These contrasting findings may reflect different time horizons, as our study similarly showed that education and exercise therapy may be cost-effective up to 9 years with potentially enhanced outcomes associated with more intensive nonsurgical care. Furthermore, education and exercise therapy provided in the RCT was found to be cost-effective only when adjusting for baseline participant demographics (age, sex, baseline costs, and QALYs). Our study represents an advance over earlier trial-based analyses as we constructed a cohort using national registry data that accurately represents the demographics of patients who undergo TKR in Australia.

Considering the opportunity cost of freeing up financial resources that might be invested elsewhere is important. Observed QALY losses from diverting individuals with osteoarthritis to education and exercise therapy programs prior to TKR could be ameliorated by investing in prevention programs that decrease the prevalence and/or severity of knee osteoarthritis and need for future TKR (eg, weight reduction, knee injury prevention).^39,40^ Examples of successful national implementation of such population-level prevention programs are lacking. Furthermore, early savings could be invested to target longer-term behavior change known to influence osteoarthritis outcomes, including quality of life. Education and exercise therapy have a limited influence on increasing physical activity participation or body weight.^41,42^ Recent research has indicated that adding a specific dietary intervention to exercise therapy may result in greater improvement in quality of life,^5^ and weight reduction has been associated with a reduced incidence of TKR.^43^

Prioritizing nonsurgical management of knee osteoarthritis to patients for whom TKR is inappropriate may be a more efficient strategy as TKR is highly effective when used appropriately.^44^ Various TKR appropriateness tools, including those that consider preoperative pain, have been developed,^45,46,47,48^ but the cost-effectiveness of implementing them as part of a care pathway has not been assessed. We were unable to identify the proportion of inappropriate TKR procedures within the current model, and the no or mild pain subgroup should not be conflated with inappropriate procedures. Future efforts are needed to test the cost-effectiveness of valid and reliable appropriateness criteria that enable the right patient to receive the right care at the right time.

Public acceptance of nonsurgical management programs for osteoarthritis such as GLA:D, while growing in Australia and internationally,^8,49^ is unclear at this end point of the care pathway. Previous studies have revealed that some individuals may be willing to accept smaller benefits from nonsurgical management to avoid undergoing TKR.^13,50^ Similarly, Hawker et al^45^ observed that 13% to 21% of Canadians who received a TKR were not ready or willing to undergo TKR, which was associated with poor outcomes. While the generalizability of this finding is unclear, public acceptance and cost-effectiveness may be improved by targeting nonsurgical management to individuals willing to delay or avoid TKR and accept smaller quality-of-life benefits than might be gained from surgery.

### Limitations

This study has several limitations. We recognize that the cost-effectiveness of a structured education and exercise therapy program may have been underestimated by not incorporating other benefits. Waiting for TKR may be associated with quality-of-life deterioration^51^ and the substantial health care investment needed to meet future demand.^2^ Not incorporating the 30% of individuals waiting more than 1 year for TKR in Australian public hospitals may overestimate the cost-effectiveness of TKR.^52^ Surgery is also associated with substantial work and productivity losses in the short term,^53^ which have not been incorporated into cost-effectiveness estimates. It is unclear whether education and exercise therapy programs such as GLA:D are positively associated with return to work and productivity. Further analyses from a societal perspective (eg, examining employment absenteeism, presenteeism, early retirement, and caregiver costs) may be warranted.

Our cohort may not be representative of patients with knee osteoarthritis in other health care systems (eg, primary care) or other countries. Despite restricting analysis of the GLA:D registry to individuals considered eligible for TKR, it is unclear whether this population is similar (given unmeasured factors) to those who undergo surgery. We did not have access to imaging data and recognize that radiographic changes can be used for TKR decision making.^6,47,48^ Furthermore, individuals in the GLA:D Australia Registry reported higher quality of life at baseline compared with those in the AOANJRR. We accounted for this baseline difference as outlined in the Methods. We acknowledge current uncertainty related to the outcomes of nonsurgical management in TKR avoidance, especially over the long term. The rate of TKR avoidance was derived from 2-year follow-up of the only published RCT,^10^ and imprecision in this estimate may substantially influence the outcomes. Furthermore, the original trial excluded individuals with severe pain (>60 mm on a 100-mm visual analog pain scale), limiting the applicability of this model input for those with severe or extreme pain at baseline. We assumed a 12-week structured education and exercise therapy program based on the original trial ,^10^ but it is unclear what the most efficient version of this program may be, as a 6-week program has been implemented in Australia with positive outcomes^8^ and therapeutic exercise may only have a small overall influence on pain and physical function.^54^ Finally, the rate of TKR following education and exercise therapy was applied equally across health states and age and sex strata given a lack of stratified data on progression to TKR.

## Conclusions

In this economic analysis, the findings suggest that structured education and exercise therapy may be cost-effective in the first 9 years but not over a lifetime horizon. The cost-effectiveness of these programs might be improved by targeting implementation to specific patient subgroups, particularly individuals with lower preoperative pain levels. These findings highlight the need to prioritize tools that identify individuals for whom nonsurgical treatment is the preferred strategy and suggest that such tools may deliver substantial cost savings and improve timely access and the quality of care for those with knee osteoarthritis.

## Notes

Supplement 1. **eTable 1.** Cohort Size Eligible for TKR **eTable 2.** Summary Demographics of AOANJR and GLA:D Registry Data **eTable 3.** Baseline Proportions for Each Health State **eTable 4.** Rate of All-Cause Mortality by Age and Sex Strata **eTable 5.** Rate of Excess Mortality Following Primary TKR Adjusted for Age and Sex **eTable 6.** Transition Probabilities (95% CI) Between Health States Following TKR **eTable 7.** Transition Probabilities Between Health States (95% CI) Following Nonsurgical Management **eTable 8.** Probability (95% CI) of TKR Revision Following TKR **eTable 9.** Baseline Utilities **eTable 10.** Utilities Following Primary TKR **eTable 11.** Incremental Utilities/Disutilities Following Nonsurgical Management Based on Transition Between Health States **eTable 12.** Total (First Row) and Per-Person (Second Row) Costs, Utilities, and Net Monetary Benefit Over the Follow-Up Period **eFigure.** Tornado Chart Summarizing Deterministic Sensitivity Analysis for Selected Variables
eReferences

Supplement 2.
Data Sharing Statement
