# Supplementary material for: Lifetime Cost-Effectiveness of Structured Education and Exercise Therapy for Knee Osteoarthritis in Australia
Source: JAMA Netw Open. 2024 Oct 1;7(10):e2436715. doi: 10.1001/jamanetworkopen.2024.36715 (PMC11445685; doi:10.1001/jamanetworkopen.2024.36715)
Supplement: Supplement 1. — eTable 1. Cohort Size Eligible for TKR eTable 2. Summary Demographics of AOANJR and GLA:D Registry Data eTable 3. Baseline Proportions for Each Health State eTable 4. Rate of All-Cause Mortality by Age and Sex Strata eTable 5. Rate of Excess Mortality Following Primary TKR Adjusted for Age and Sex eTable 6. Transition Probabilities (95% CI) Between Health States Following TKR eTable 7. Transition Probabilities Between Health States (95% CI) Following Nonsurgical Management eTable 8. Probability (95% CI) of TKR Revision Following TKR eTable 9. Baseline Utilities eTable 10. Utilities Following Primary TKR eTable 11. Incremental Utilities/Disutilities Following Nonsurgical Management Based on Transition Between Health States eTable 12. Total (First Row) and Per-Person (Second Row) Costs, Utilities, and Net Monetary Benefit Over the Follow-Up Period eFigure. Tornado Chart Summarizing Deterministic Sensitivity Analysis for Selected Variables eReferences [file jamanetwopen-e2436715-s001.pdf]

Supplemental Online Content

Docking S, Ademi Z, Barton C, et al. Lifetime cost-effectiveness of structured education and exercise therapy for knee osteoarthritis in Australia. *JAMA Netw Open.* 2024;7(10):e2436715. doi:10.1001/jamanetworkopen.2024.36715

eTable 1. Cohort Size Eligible for TKR

eTable 2. Summary Demographics of AOANJR and GLA:D Registry Data

eTable 3. Baseline Proportions for Each Health State

eTable 4. Rate of All-Cause Mortality by Age and Sex Strata

eTable 5. Rate of Excess Mortality Following Primary TKR Adjusted for Age and Sex

eTable 6. Transition Probabilities (95% CI) Between Health States Following TKR

eTable 7. Transition Probabilities Between Health States (95% CI) Following Nonsurgical Management

eTable 8. Probability (95% CI) of TKR Revision Following TKR

eTable 9. Baseline Utilities

eTable 10. Utilities Following Primary TKR

eTable 11. Incremental Utilities/Disutilities Following Nonsurgical Management Based on Transition Between Health States

eTable 12. Total (First Row) and Per-Person (Second Row) Costs, Utilities, and Net Monetary Benefit Over the Follow-Up Period

eFigure. Tornado Chart Summarizing Deterministic Sensitivity Analysis for Selected Variables

eReferences

This supplemental material has been provided by the authors to give readers additional information about their work.

© 2024 Docking S et al. *JAMA Network Open.*

eTable 1. Cohort Size Eligible for TKR

	Australian population (2022)	Rate of TKR (per 100,000)	Cohort size (n)	Australian population (2022)	Rate of TKR (per 100,000)	Cohort size (n)
	Females			Males		
45-49 years	832,618	67.8	**565**	817,346	56.5	**462**
50-54 years	817,482	196.8	**1,609**	794,263	164.6	**1,307**
55-59 years	788,117	406.3	**3,202**	762,686	385.8	**2,942**
60-64 years	754,125	693.3	**5,228**	711,335	730.3	**5,195**
65-69 years	662,761	1,013.30	**6,716**	617,714	967.2	**5,975**
70-74 years	592,099	1,243.10	**7,360**	554,728	1,115.60	**6,189**
75-79 years	419,384	1,307.90	**5,485**	387,887	1,078.30	**4,183**
80-84 years	295,455	983	**2,904**	249,963	828.8	**2,072**

Source: [1,2]

© 2024 Docking S et al. *JAMA Network Open.*

eTable 2. Summary Demographics of AOANJR and GLA:D Registry Data

	AOANJRR (n = 9,889)	GLA:D® Australia (n=741)
**Age group, n (%)**		
45-54 years	698 (7.1)	93 (12.5)
55-64 years	3,019 (30.5)	283 (38.2)
65-74 years	4,368 (44.2)	296 (40.0)
75-84 years	1,804 (18.2)	69 (9.3)
**Number of females (%)**	5,508 (55.7)	485 (65.5)
**Baseline health state, n (%)**		
No/mild pain	1,952 (19.7)	178 (24.0)
Moderate pain	4,961 (50.2)	452 (61.0)
Severe/extreme pain	2,976 (30.1)	111 (15.0)
**Baseline EQ-5D-5L, mean (SD)***		
All participants	0.660 (0.251)	0.754 (0.184)
No/mild pain	0.880 (0.085)	0.887 (0.074)
Moderate pain	0.753 (0.127)	0.785 (0.097)
Severe/extreme pain	0.359 (0.205)	0.418 (0.182)

*Score range from -0.301 to 1, with higher scores representing greater quality of life

Note: While baseline EQ-5D-5L scores differed overall between registries, this was accounted for as all model inputs were stratified based on health state.

© 2024 Docking S et al. *JAMA Network Open.*

eTable 3. Baseline Proportions for Each Health State

Patient-reported outcomes data were requested and obtained from the AOANJRR. We excluded individuals who:

- Were younger than 45 years old or older than 85 years old,
- Had undergone revision TKR within a year of primary TKR,
- Had incomplete data pre- or post-operatively,
- Provided pre-operative scores after TKR or more than 3 months before TKR.

The final cohort included 9,889 individuals who had undergone primary TKR.

	No/slight pain	Moderate pain	Severe/extreme pain
	**Females; proportion (95% CI)**		
**45-54 years**	0.081 (0.058 to 0.111)	0.469 (0.422 to 0.518)	0.450 (0.402 to 0.498)
**55-64 years**	0.152 (0.135 to 0.170)	0.511 (0.487 to 0.536)	0.337 (0.314 to 0.361)
**65-74 years**	0.193 (0.177 to 0.209)	0.513 (0.493 to 0.533)	0.294 (0.276 to 0.312)
**75-84 years**	0.194 (0.172 to 0.219)	0.498 (0.468 to 0.527)	0.308 (0.281 to 0.336)
	**Males; proportion (95% CI)**		
**45-54 years**	0.111 (0.079 to 0.152)	0.453 (0.397 to 0.511)	0.436 (0.380 to 0.494)
**55-64 years**	0.176 (0.157 to 0.196)	0.502 (0.477 to 0.529)	0.322 (0.298 to 0.347)
**65-74 years**	0.249 (0.231 to 0.269)	0.508 (0.486 to 0.530)	0.243 (0.224 to 0.262)
**75-84 years**	0.322 (0.289 to 0.357)	0.466 (0.430 to 0.503)	0.212 (0.184 to 0.244)

© 2024 Docking S et al. *JAMA Network Open.*

eTable 4. Rate of All-Cause Mortality by Age and Sex Strata

Age group	Females (per 100,000)	Males (per 100,000)
**45-54 years**	153	259
**55-64 years**	345	585
**65-74 years**	804	1,327
**75-84 years**	2,532	3,798
**85-94 years**	9,863	12,815
**95-100 years**	24,767	27,018

Standard error ±25% was incorporated in sensitivity analysis.

Source: [3]

© 2024 Docking S et al. *JAMA Network Open.*

eTable 5. Rate of Excess Mortality Following Primary TKR Adjusted for Age and Sex

Years post primary TKR	Excess mortality (95% CI)
**1**	-0.012 (-0.013 to -0.012)
**2**	-0.011 (-0.012 to -0.011)
**3**	-0.01 (-0.011 to -0.01)
**4**	-0.009 (-0.01 to -0.008)
**5**	-0.008 (-0.009 to -0.008)
**6**	-0.007 (-0.008 to -0.006)
**7**	-0.005 (-0.006 to -0.005)
**8**	-0.005 (-0.006 to -0.004)
**9**	-0.002 (-0.004 to -0.001)
**10**	0.0001 (-0.002 to 0.0016)
**11**	0.0012 (-0.001 to 0.0031)
**12**	0.0036 (0.0011 to 0.0061)
**13**	0.0051 (0.0016 to 0.0086)

Source: [4]

© 2024 Docking S et al. *JAMA Network Open.*

eTable 6. Transition Probabilities (95% CI) Between Health States Following TKR

Females 45-54 years old		Post-TKR		
Pre-TKR		No/slight	Moderate	Severe/extreme
	No/slight	0.8788 (0.71 to 0.956)	0.1212 (0.045 to 0.29)	0 (0 to 0.104)
	Moderate	0.7604 (0.695 to 0.816)	0.224 (0.17 to 0.289)	0.0156 (0.005 to 0.048)
	Severe/extreme	0.6902 (0.619 to 0.753)	0.1957 (0.144 to 0.26)	0.1141 (0.075 to 0.169)
Females 55-64 years old		Post-TKR		
Pre-TKR		No/slight	Moderate	Severe/extreme
	No/slight	0.9054 (0.861 to 0.936)	0.0864 (0.057 to 0.129)	0.0082 (0.002 to 0.033)
	Moderate	0.7863 (0.757 to 0.813)	0.1844 (0.159 to 0.212)	0.0293 (0.02 to 0.043)
	Severe/extreme	0.6407 (0.599 to 0.68)	0.2463 (0.212 to 0.285)	0.113 (0.089 to 0.146)
Females 65-74 years old		Post-TKR		
Pre-TKR		No/slight	Moderate	Severe/extreme
	No/slight	0.9097 (0.88 to 0.933)	0.086 (0.064 to 0.115)	0.0043 (0.001 to 0.017)
	Moderate	0.8052 (0.782 to 0.826)	0.1705 (0.151 to 0.193)	0.0243 (0.017 to 0.035)
	Severe/extreme	0.6822 (0.647 to 0.716)	0.2401 (0.21 to 0.273)	0.0777 (0.06 to 0.1)
Females 75-84 years old		Post-TKR		
Pre-TKR		No/slight	Moderate	Severe/extreme
	No/slight	0.8389 (0.783 to 0.883)	0.1327 (0.093 to 0.186)	0.0284 (0.013 to 0.062)
	Moderate	0.817 (0.782 to 0.847)	0.1479 (0.12 to 0.18)	0.0351 (0.022 to 0.054)
	Severe/extreme	0.6866 (0.635 to 0.734)	0.2209 (0.179 to 0.269)	0.0925 (0.066 to 0.129)
Males 45-54 years old		Post-TKR		
Pre-TKR		No/slight	Moderate	Severe/extreme
	No/slight	0.8437 (0.667 to 0.936)	0.125 (0.046 to 0.298)	0.0313 (0.004 to 0.204)
	Moderate	0.8397 (0.766 to 0.894)	0.1374 (0.088 to 0.208)	0.0229 (0.007 to 0.069)
	Severe/extreme	0.746 (0.662 to 0.815)	0.2222 (0.158 to 0.304)	0.0318 (0.012 to 0.082)

© 2024 Docking S et al. *JAMA Network Open.*

Males 55-64 years old		Post-TKR		
Pre-TKR		No/slight	Moderate	Severe/extreme
	No/slight	0.9077 (0.965 to 0.938)	0.0843 (0.056 to 0.126)	0.008 (0.002 to 0.032)
	Moderate	0.7809 (0.749 to 0.81)	0.1882 (0.161 to 0.219)	0.0309 (0.02 to 0.047)
	Severe/extreme	0.7127 (0.67 to 0.753)	0.2412 (0.204 to 0.283)	0.0461 (0.03 to 0.07)
Males 65-74 years old		Post-TKR		
Pre-TKR		No/slight	Moderate	Severe/extreme
	No/slight	0.9037 (0.874 to 0.927)	0.0881 (0.066 to 0.117)	0.0082 (0.003 to 0.022)
	Moderate	0.7739 (0.747 to 0.799)	0.205 (0.181 to 0.231)	0.0211 (0.014 to 0.032)
	Severe/extreme	0.7516 (0.711 to 0.788)	0.1874 (0.155 to 0.225)	0.061 (0.043 to 0.087)
Males 75-84 years old		Post-TKR		
Pre-TKR		No/slight	Moderate	Severe/extreme
	No/slight	0.8138 (0.758 to 0.859)	0.1602 (0.118 to 0.214)	0.026 (0.012 to 0.057)
	Moderate	0.7964 (0.75 to 0.836)	0.1767 (0.139 to 0.221)	0.0269 (0.014 to 0.051)
	Severe/extreme	0.6776 (0.599 to 0.748)	0.2303 (0.17 to 0.304)	0.0921 (0.055 to 0.15)

© 2024 Docking S et al. *JAMA Network Open.*

eTable 7. Transition Probabilities Between Health States (95% CI) Following Nonsurgical Management

Data was requested and obtained from the GLA:D® Australia registry [5]. We excluded individuals who:

- Were younger than 45 years old or older than 85 years old,
- Had previously undergone surgery primary TKR,

We included individuals who:

- Were on a waiting list for a joint replacement or surgical opinion, or
- Reported so much trouble and pain that they want surgery, or
- Had a KOOS-12 summary score equal or greater that 54.6.

The final cohort included 741 individuals who had received the GLA:D® non-surgical management program.

Females 45-54 years old		Post non-surgical		
		**No/slight**	**Moderate**	**Severe/extreme**
Pre non-surgical	**No/slight**	0.7777 (0.514 to 0.92)	0.1667 (0.05 to 0.432)	0.0556 (0.007 to 0.34)
	**Moderate**	0.4545 (0.312 to 0.606)	0.4546 (0.312 to 0.606)	0.0909 (0.034 to 0.224)
	**Severe/extreme**	0.1111 (0.011 to 0.591)	0.2222 (0.043 to 0.645)	0.6667 (0.281 to 0.911)
**Females 55-64 years old**		**Post non-surgical**		
		**No/slight**	**Moderate**	**Severe/extreme**
Pre non-surgical	**No/slight**	0.8 (0.54 to 0.894)	0.1778 (0.09 to 0.322)	0.0222 (0.003 to 0.149)
	**Moderate**	0.537 (0.442 to 0.63)	0.3519 (0.267 to 0.447)	0.1111 (0.064 to 0.187)
	**Severe/extreme**	0.3415 (0.21 to 0.502)	0.4146 (0.272 to 0.573)	0.2439 (0.134 to 0.402)
**Females 65-74 years old**		**Post non-surgical**		
		**No/slight**	**Moderate**	**Severe/extreme**
Pre non-surgical	**No/slight**	0.8182 (0.642 to 0.919)	0.1818 (0.081 to 0.358)	0 (0 to 0.104)
	**Moderate**	0.595 (0.505 to 0.68)	0.3389 (0.26 to 0.428)	0.0661 (0.033 to 0.128)
	**Severe/extreme**	0.4815 (0.296 to 0.672)	0.2222 (0.099 to 0.425)	0.2963 (0.15 to 0.5)
**Females 75-84 years old**		**Post non-surgical**		
		**No/slight**	**Moderate**	**Severe/extreme**
Pre non-surgical	**No/slight**	0.7273 (0.371 to 0.923)	0.2727 (0.077 to 0.63)	0 (0 to 0.259)
	**Moderate**	0.5217 (0.315 to 0.722)	0.3913 (0.21 to 0.609)	0.087 (0.02 to 0.307)
	**Severe/extreme**	0.4 (0.05 to 0.894)	0.4 (0.05 to 0.894)	0.2 (0.011 to 0.848)

© 2024 Docking S et al. *JAMA Network Open.*

Males 45-54 years old		Post non-surgical		
Pre non-surgical		No/slight	Moderate	Severe/extreme
	No/slight	0.8 (0.152 to 0.989)	0.2 (0.011 to 0.848)	0 (0 to 0.434)
	Moderate	0.5 (0.219 to 0.781)	0.4167 (0.164 to 0.722)	0.0833 (0.009 to 0.475)
	Severe/extreme	0.2 (0.011 to 0.848)	0.4 (0.05 to 0.894)	0.4 (0.05 to 0.894)
Males 55-64 years old		Post non-surgical		
Pre non-surgical		No/slight	Moderate	Severe/extreme
	No/slight	0.8 (0.554 to 0.928)	0.2 (0.072 to 0.446)	0 (0 to 0.161)
	Moderate	0.4815 (0.35 to 0.616)	0.5 (0.367 to 0.633)	0.0185 (0.003 to 0.125)
	Severe/extreme	0.2 (0.06 to 0.5)	0.6 (0.326 to 0.823)	0.2 (0.06 to 0.5)
Males 65-74 years old		Post non-surgical		
Pre non-surgical		No/slight	Moderate	Severe/extreme
	No/slight	0.8333 (0.669 to 0.925)	0.1667 (0.075 to 0.331)	0 (0 to 0.096)
	Moderate	0.4658 (0.353 to 0.582)	0.4383 (0.328 to 0.555)	0.0959 (0.046 to 0.19)
	Severe/extreme	0.6667 (0.178 to 0.949)	0.1667 (0.012 to 0.77)	0.1666 (0.012 to 0.77)
Females 75-84 years old		Post non-surgical		
Pre non-surgical		No/slight	Moderate	Severe/extreme
	No/slight	0.9 (0.453 to 0.99)	0.1 (0.01 to 0.547)	0 (0 to 0.278)
	Moderate	0.4706 (0.241 to 0.714)	0.4118 (0.198 to 0.666)	0.1176 (0.026 to 0.397)
	Severe/extreme	0.3333 (0.003 to 0.99)	0.6667 (0.01 to 0.997)	0 (0 to 0.561)

© 2024 Docking S et al. *JAMA Network Open.*

eTable 8. Probability (95% CI) of TKR Revision Following TKR

	Rate of revision TKR (95% CI)			
	**Females**			
**Years post primary TKR**	**45-54 years old**	**55-64 years old**	**65-74 years old**	**75-84 years old**
**1**	0.013 (0.012 to 0.015)	0.009 (0.009 to 0.01)	0.007 (0.007 to 0.008)	0.007 (0.007 to 0.008)
**3**	0.04 (0.038 to 0.043)	0.027 (0.026 to 0.028)	0.021 (0.02 to 0.022)	0.016 (0.015 to 0.016)
**5**	0.056 (0.053 to 0.059)	0.037 (0.036 to 0.038)	0.028 (0.027 to 0.028)	0.02 (0.019 to 0.021)
**10**	0.087 (0.082 to 0.091)	0.057 (0.055 to 0.059)	0.04 (0.039 to 0.041)	0.027 (0.026 to 0.028)
**15**	0.123 (0.116 to 0.13)	0.08 (0.077 to 0.083)	0.05 (0.048 to 0.052)	0.032 (0.03 to 0.034)
**20**	0.159 (0.146 to 0.173)	0.101 (0.095 to 0.108)	0.059 (0.055 to 0.063)	0.033 (0.031 to 0.036)
	**Males**			
	**45-54 years old**	**55-64 years old**	**65-74 years old**	**75-84 years old**
**1**	0.019 (0.017 to 0.021)	0.013 (0.012 to 0.014)	0.011 (0.011 to 0.012)	0.01 (0.009 to 0.011)
**3**	0.048 (0.045 to 0.051)	0.032 (0.031 to 0.034)	0.025 (0.024 to 0.026)	0.02 (0.019 to 0.021)
**5**	0.062 (0.058 to 0.066)	0.042 (0.41 to 0.043)	0.033 (0.032 to 0.034)	0.024 (0.023 to 0.026)
**10**	0.093 (0.088 to 0.098)	0.063 (0.061 to 0.065)	0.046 (0.045 to 0.048)	0.032 (0.03 to 0.033)
**15**	0.136 (0.128 to 0.145)	0.087 (0.084 to 0.09)	0.058 (0.056 to 0.061)	0.037 (0.035 to 0.04)
**20**	0.175 (0.16 to 0.192)	0.11 (0.103 to 0.118)	0.067 (0.063 to 0.071)	0.038 (0.035 to 0.041)

Source: Table KT16 on page 212 in [6]

© 2024 Docking S et al. *JAMA Network Open.*

eTable 9. Baseline Utilities

	No/slight pain	Moderate pain	Severe/extreme pain
**Females; mean (95% CI)**			
**45-54 years**	0.861 (0.821 to 0.901)	0.744 (0.726 to 0.761)	0.346 (0.316 to 0.376)
**55-64 years**	0.868 (0.854 to 0.882)	0.749 (0.740 to 0.758)	0.331 (0.312 to 0.349)
**65-74 years**	0.883 (0.877 to 0.890)	0.764 (0.757 to 0.770)	0.368 (0.353 to 0.383)
**75-84 years**	0.873 (0.861 to 0.885)	0.748 (0.736 to 0.760)	0.350 (0.327 to 0.373)
Males; mean (95% CI)			
**45-54 years**	0.880 (0.856 to 0.904)	0.728 (0.706 to 0.750)	0.375 (0.341 to 0.409)
**55-64 years**	0.880 (0.870 to 0.890)	0.746 (0.737 to 0.756)	0.369 (0.351 to 0.386)
**65-74 years**	0.885 (0.878 to 0.891)	0.759 (0.751 to 0.767)	0.375 (0.357 to 0.392)
**75-84 years**	0.880 (0.868 to 0.893)	0.744 (0.731 to 0.758)	0.367 (0.336 to 0.398)

Utilities represented on scale from -0.301 to 1, with 1 representing full health

© 2024 Docking S et al. *JAMA Network Open.*

eTable 10. Utilities Following Primary TKR

	No/slight pain	Moderate pain	Severe/extreme pain
**Females; mean (95% CI)**			
**45-54 years**	0.929 (0.921 to 0.937)	0.761 (0.734 to 0.788)	0.278 (0.171 to 0.385)
**55-64 years**	0.937 (0.933 to 0.940)	0.766 (0.752 to 0.780)	0.303 (0.253 to 0.353)
**65-74 years**	0.941 (0.938 to 0.944)	0.790 (0.779 to 0.801)	0.388 (0.346 to 0.430)
**75-84 years**	0.937 (0.932 to 0.942)	0.777 (0.758 to 0.795)	0.385 (0.323 to 0.446)
Males; mean (95% CI)			
**45-54 years**	0.939 (0.931 to 0.946)	0.744 (0.712 to 0.776)	0.341 (0.094 to 0.589)
**55-64 years**	0.936 (0.932 to 0.940)	0.771 (0.757 to 0.785)	0.332 (0.266 to 0.399)
**65-74 years**	0.941 (0.938 to 0.944)	0.773 (0.760 to 0.786)	0.368 (0.300 to 0.435)
**75-84 years**	0.940 (0.933 to 0.946)	0.782 (0.759 to 0.806)	0.328 (0.260 to 0.396)

Utilities represented on scale from -0.301 to 1, with 1 representing full health

© 2024 Docking S et al. *JAMA Network Open.*

eTable 11. Incremental Utilities/Disutilities Following Nonsurgical Management Based on Transition Between Health States

		Post non-surgical; mean (95% CI)		
		**No/slight**	**Moderate**	**Severe/extreme**
Pre non-surgical	**No/slight**	0.039 (-0.039 to 0.123)	-0.075 (-0.326 to 0.034)	-0.555 (-0.592 to -0.517)
	**Moderate**	0.128 (0.037 to 0.322)	0.006 (-0.17 to 0.192)	-0.357 (-0.555 to -0.11)
	**Severe/extreme**	0.458 (0.29 to 0.727)	0.389 (0.14 to 0.704)	-0.024 (-0.318 to 0.33)

© 2024 Docking S et al. *JAMA Network Open.*

eTable 12. Total (First Row) and Per-Person (Second Row) Costs, Utilities, and Net Monetary Benefit Over the follow-up period

	Costs ($AUD 2022)			QALYs			
	Non-surgical	Usual care	Difference	Non-surgical	Usual care	Difference	NMB
1	$260 456,234	$1,441,589,864	-$1,181,133,631	42,470	44,421	- 1,950	$18,348
	$4,242	$23,481	-$19,229	0.69	0.72	- 0.03	
3	$570,816,471	$1,476,973,874	-$906,157,403	128,435	137,982	- 9,548	$10,400
	$9,298	$24,057	-$14,760	2.09	2.25	- 0.16	
5	$704,769,244	$1,498,527,727	-$793,758,483	205,563	220,615	- 15,052	$6,056
	$11,479	$24,408	-$12,929	3.35	3.59	- 0.25	
10	$906,303,078	$1,523,910,258	-$617,607,180	364,358	387,282	- 22,924	-$408
	$14,762	$24,822	-$10,060	5.93	6.31	- 0.37	
20	$1,048,463,674	$1,553,794,733	-$505,331,059	545,734	573,543	- 27,351	-$4,010
	$17,078	$25,309	-$8,231	8.91	9.34	- 0.44	
30	$1,081,149,257	$1,570,844,921	-$489,695,663	609,898	636,589	- 26,691	-$4,210
	$17,610	$25,586	-$7,976	9.93	10.37	- 0.43	
40	$1,090,458,832	$1,579,588,785	-$489,129,953	623,126	649,586	-26,460	-$4,115
	$17,762	$25,729	-$7,967	10.15	10.58	- 0.43	
50	$1,092,713,293	$1,582,021,235	-$489,307,942	624,489	650,902	- 26,412	-$4,090
	$17,798	$25,768	-$7,970	10.17	10.60	- 0.43	

© 2024 Docking S et al. *JAMA Network Open.*

eFigure. Tornado Chart Summarizing Deterministic Sensitivity Analysis for Selected Variables

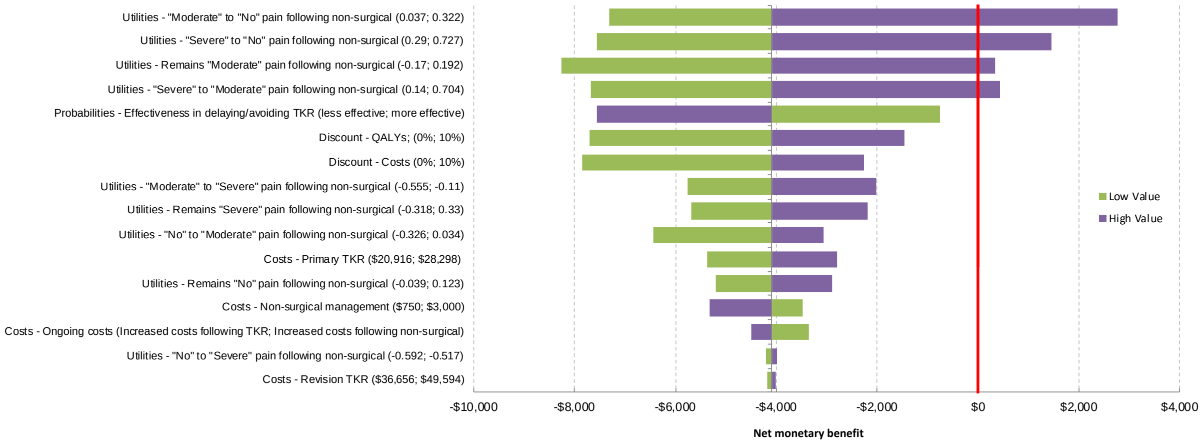

© 2024 Docking S et al. *JAMA Network Open.*

eReferences

1. Australian Bureau of Statistics. *National, State and Territory Population*. ABS; 2022. https://www.abs.gov.au/statistics/people/population/national-state-and-territory-population/latest-release

2. Australian Institute of Health and Welfare; *Chronic Musculoskeletal Conditions*. AIHW; 2023. https://www.aihw.gov.au/reports/chronic-musculoskeletal-conditions/musculoskeletal-conditions

3. Australian Bureau of Statistics. *Life Tables, 2019-2021*. ABS; 2023. https://www.abs.gov.au/statistics/people/population/life-tables/latest-release

4. Harris IA, Hatton A, Pratt N, et al. How Does Mortality Risk Change Over Time After Hip and Knee Arthroplasty? *Clin Orthop Relat Res*. 2019;477(6):1414-1421. doi:10.1097/CORR.0000000000000673

5. Barton CJ, Kemp JL, Roos EM, et al. Program evaluation of GLA:D® Australia: Physiotherapist training outcomes and effectiveness of implementation for people with knee osteoarthritis. *Osteoarthr Cartil Open*. 2021;3(3):100175. doi:10.1016/j.ocarto.2021.100175

6. Australian Orthopaedic Association National Joint Replacement Registry (AOANJRR). *Hip, Knee & Shoulder Arthroplasty: 2022 Annual Report*.; 2022. https://aoanjrr.sahmri.com/annual-reports-2022

© 2024 Docking S et al. *JAMA Network Open.*
