# Supplementary material for: Lifetime Cost-Effectiveness of Structured Education and Exercise Therapy for Knee Osteoarthritis in Australia
Source: JAMA Netw Open. 2024 Oct 1;7(10):e2436715. doi: 10.1001/jamanetworkopen.2024.36715 (PMC11445685; doi:10.1001/jamanetworkopen.2024.36715)
Supplement: Supplement 2. — Data Sharing Statement [file jamanetwopen-e2436715-s002.pdf]

Data Sharing Statement

Docking. Lifetime Cost-Effectiveness of Structured Education and Exercise Therapy for Knee Osteoarthritis in Australia. *JAMA Netw Open*. Published October 01, 2024. doi:10.1001/jamanetworkopen.2024.36715

Data
Data available: No
